# Diagnosis of small bowel obstruction due to Shine-Muscat grape ingestion: case report

**DOI:** 10.3389/fped.2024.1503456

**Published:** 2024-12-19

**Authors:** Chunhui Gu, Youcheng Zhang, Guoqing Jiang, Xiaoting Hu

**Affiliations:** ^1^ Huai’an Maternal and Child Health Care Hospital Affiliated to Yangzhou University, Huai’an, China; ^2^ Hepatobiliary Surgery, Northern Jiangsu People's Hospital Affiliated to Yangzhou University, Yangzhou, China

**Keywords:** small bowel obstruction, Shine-Muscat grape ingestion, foreign body, pediatric, laparoscopic surgery

## Abstract

**Introduction:**

This case report describes a rare instance of small bowel obstruction (SBO) caused by the ingestion of a whole Shine-Muscat grape in a 7-month-old infant. This case adds to the scientific literature by highlighting the potential risk of common fruits, such as grapes, in causing serious gastrointestinal blockages in pediatric patients, which is an uncommon but important consideration for pediatricians and caregivers.

**Main symptoms and clinical findings:**

A 7-month-old female presented with a 3-day history of vomiting, which progressed to bilious vomiting, accompanied by abdominal distension and dehydration. Abdominal CT imaging revealed dilated small bowel loops and a spherical low-density lesion suggestive of an obstructing foreign body.

**Main diagnoses, therapeutic interventions, and outcomes:**

The diagnosis of mechanical small bowel obstruction due to a foreign body was confirmed intraoperatively. The obstructing object was identified as a whole Shine-Muscat grape. Surgical management involved a minimally invasive laparoscopic approach to crush and move the grape into the colon, avoiding bowel incision. The patient recovered well postoperatively and passed the grape fragments naturally, resuming a normal diet within days.

**Conclusion:**

This case underscores the importance of considering fruit ingestion as a potential cause of intestinal obstruction in infants. Pediatric surgeons and emergency clinicians should be aware of this rare but significant risk and employ careful history-taking, appropriate imaging, and minimally invasive techniques to manage such cases effectively.

## 1 Introduction

Small bowel obstruction (SBO) in pediatric patients is a common surgical emergency, often resulting from adhesions, hernias, or congenital abnormalities (1). However, SBO caused by the ingestion of a whole grape is exceptionally rare, particularly in infants. Previous literature has primarily focused on foreign body ingestion involving objects like coins, magnets, and bezoars, with fruits being an uncommon cause (2–7). This case report presents a unique instance of SBO in a 7-month-old infant due to the ingestion of a whole Shine-Muscat grape, a situation not well-documented in current medical literature.

This case adds to the understanding of pediatric gastrointestinal emergencies by highlighting the need for increased awareness among pediatricians and caregivers regarding the potential hazards of feeding whole fruits to young children. It emphasizes the importance of thorough history-taking, careful radiological evaluation, and consideration of minimally invasive surgical techniques for effective management. By documenting this rare occurrence, we aim to provide valuable insights for clinical practice and preventive strategies to reduce the risk of such potentially life-threatening events in pediatric patients.

## 2 Case presentation

### 2.1 Patient information

A 7-month-old female infant presented to the emergency department with a primary concern of repeated vomiting over 3 days, which progressed to bilious vomiting on the final day. The parents also reported abdominal distension and signs of dehydration. There was no history of fever or abdominal pain reported, and the parents specifically denied any prior instances of foreign body ingestion. The infant had no significant medical, family, or psychosocial history, including no known genetic disorders. There were no relevant past surgical interventions or conditions.

### 2.2 Clinical findings

On physical examination, the infant appeared dehydrated with abdominal distension but was afebrile and showed no signs of abdominal tenderness or palpable masses. No surgical scars were present on the abdomen, and no bilateral inguinal hernias were detected. The rectal examination revealed no fecal matter, and a corkscrew enema failed to induce bowel movement. Additionally, abdominal x-ray was performed, which revealed characteristic air-fluid levels, further supporting the diagnosis. Given these findings, a diagnosis of mechanical small bowel obstruction was suspected.

### 2.3 Timeline

Day 1: The infant began experiencing episodes of non-bilious vomiting and mild abdominal distension. Symptoms were initially intermittent and mild, with no other associated signs such as fever or abdominal pain.

Day 2: Vomiting episodes continued, becoming more frequent and severe. Abdominal distension worsened, and the infant showed signs of decreased oral intake and activity levels. No bowel movements were reported.

**Figure 1:**
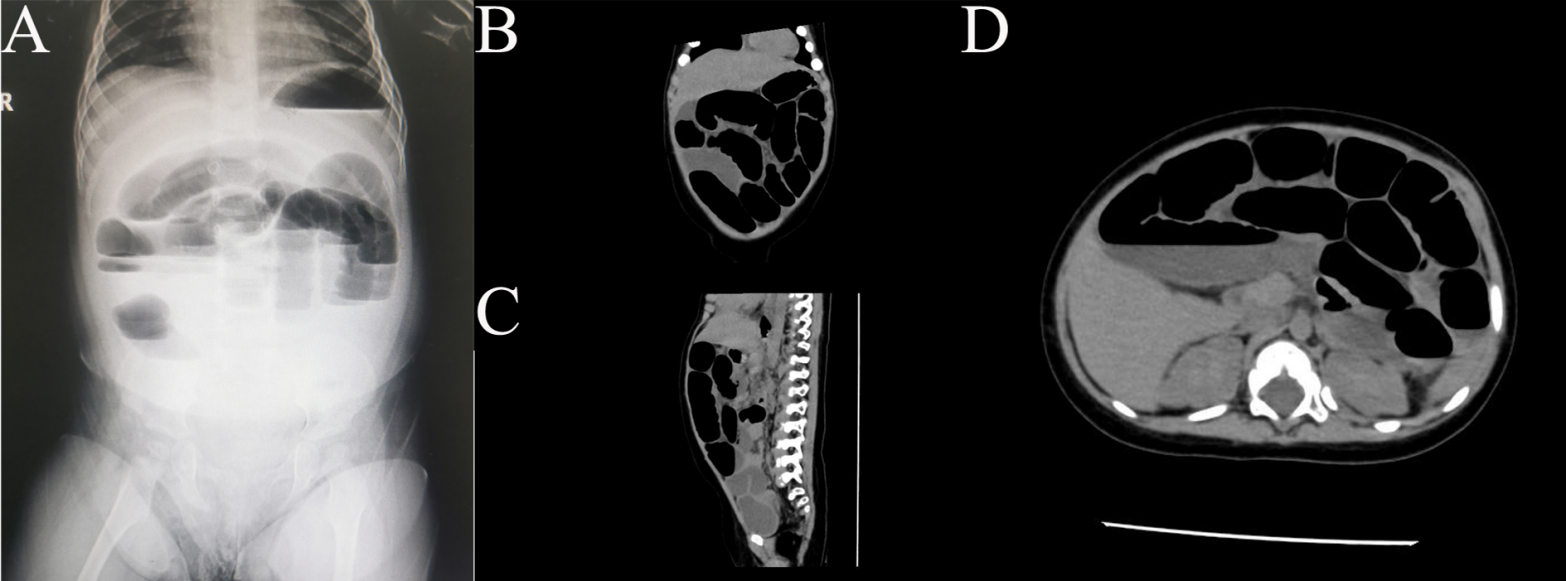
Imaging findings of small bowel obstruction Due to Shine-Muscat grape in an infant. **(A)** An upright abdominal radiograph of the infant showing signs of small bowel obstruction, characterized by multiple dilated loops of bowel and air-fluid levels suggestive of a mechanical obstruction. **(B–D)** Abdominal CT scans in various planes (sagittal, coronal, and axial) further confirm the presence of small bowel obstruction. The images demonstrate dilated intestinal loops with a transition point, suggesting a luminal obstruction caused by the ingestion of a Shine-Muscat grape. No evidence of perforation or additional masses is observed.

Day 3: The vomiting turned bilious, and the infant developed significant abdominal distension, prompting the parents to bring her to the emergency department. On examination, the patient appeared dehydrated but was afebrile, with notable abdominal distension. Initial diagnostic workup, including an abdominal x-ray, was performed, revealing step-like air-fluid levels suggestive of small bowel obstruction (Figure 1A).

**Figure 2:**
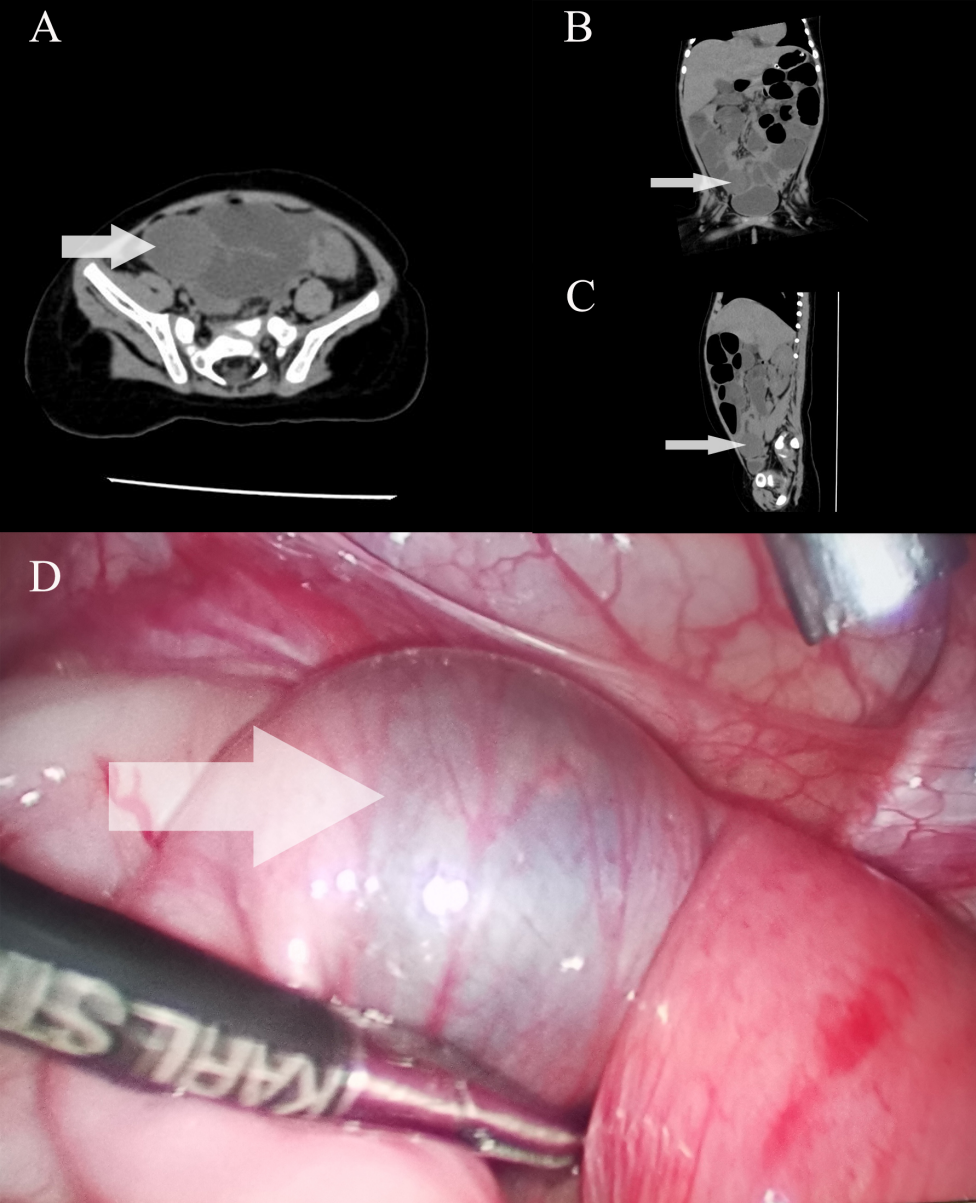
Imaging and intraoperative findings of Shine-Muscat grape-induced small bowel obstruction in an infant. Abdominal CT scans in axial **(A)**, coronal **(B)**, and sagittal **(C)** planes, clearly showing the location of the foreign body (Shine-Muscat grape) causing small bowel obstruction. The arrows indicate the exact position of the grape in the intestinal lumen, which appears as a well-defined round mass causing luminal narrowing and obstruction. **(D)** Intraoperative view of the foreign body located within the terminal ileum. The image shows a visibly distended proximal bowel loop (marked by the arrow), while the distal bowel loops appear collapsed. The obstruction was relieved after the surgical removal of the Shine-Muscat grape, which was intact and causing a mechanical blockage.

Day 4: A follow-up abdominal CT scan showed dilated small bowel loops with a spherical low-density lesion, consistent with an obstructive foreign body (Figures 1B–D, 2A–C). While the imaging clearly indicated the presence of a foreign body in the right lower quadrant within the ileum, it could not precisely determine the exact position of the object. Despite the parents denying any history of foreign body ingestion, the clinical and imaging findings indicated mechanical small bowel obstruction, necessitating surgical intervention. The patient underwent diagnostic laparoscopy, which confirmed the presence of a whole grape located in the terminal ileum, approximately 80 cm from the ileocecal valve. The obstruction was successfully managed laparoscopically by crushing the grape and advancing it into the colon to avoid an intestinal incision (Figure 2D).

Postoperative Days 1–2: The infant was managed with fasting, gastrointestinal decompression, and intravenous fluids. A nasogastric tube was placed in the emergency department prior to surgery and remained in place postoperatively to ensure effective decompression until gastrointestinal function was restored. During the surgery, additional decompression was achieved by using atraumatic grasping forceps to crush the foreign body and advance it towards the cecum, which contributed to the early recovery of lower gastrointestinal function. By the second postoperative day, the infant naturally passed grape fragments, and her gastrointestinal function gradually returned to normal.

Postoperative Day 5: The patient was discharged from the hospital with stable vital signs, normal diet tolerance, and no further signs of obstruction or complications. Follow-up showed no recurrence of symptoms.

### 2.4 Diagnostic assessment

Initial diagnostic workup included laboratory tests and imaging studies. The laboratory results showed an elevated C-reactive protein (CRP) level of 19.6 mg/L (normal <5 mg/L) and a procalcitonin level of 0.97 ng/ml (normal <0.5 ng/ml), indicating a potential inflammatory or infectious process. Imaging included an upright abdominal x-ray, which revealed step-like air-fluid levels, suggestive of small bowel obstruction (Figure 1A). An abdominal CT scan further confirmed the presence of dilated small bowel loops with multiple air-fluid levels in the left mid-upper abdomen (Figure 1B–D), along with a spherical low-density lesion measuring approximately 2.8 cm by 2.4 cm in the right lower abdomen, which was suspected to be a foreign body (Figure 2A–C). Despite the parents' denial of foreign body ingestion, the imaging findings raised a strong suspicion of this etiology. Differential diagnoses, including intussusception, volvulus, and infectious or inflammatory bowel disease, were considered but deemed less likely due to the clinical presentation and imaging findings. Ultimately, the diagnosis of mechanical small bowel obstruction caused by a foreign body was confirmed intraoperatively (Figure 2D).

### 2.5 Therapeutic intervention

The patient underwent an emergency diagnostic laparoscopy. A transition point in the ileum, approximately 80 cm from the ileocecal valve, was identified with proximal dilation and distal decompression. Under laparoscopic guidance, a 5 mm trocar was inserted into the abdominal cavity to introduce atraumatic grasping forceps, which were used to locate and externally crush a soft spherical foreign object through the bowel wall. The object was later identified as a Shine-Muscat grape. During the surgery, after identifying the foreign body, the parents were questioned again, and they confirmed recent grape ingestion. Based on this information, we decided to crush the grape and push it into the colon rather than making an intestinal incision, which minimized surgical trauma and facilitated a quicker recovery. Postoperatively, the patient received gastrointestinal decompression, infection prevention with antibiotics, and rehydration therapy.

### 2.6 Follow-up and outcomes

The infant showed rapid postoperative recovery, with gastrointestinal function restoring within 2 days. By the second day, she passed grape fragments naturally, and a normal diet was gradually reintroduced. By the fifth day, the patient was discharged with no significant abnormalities in routine blood tests. Follow-up after discharge confirmed the absence of any recurrent symptoms or complications, and the parents were advised on safe feeding practices to prevent future incidents of foreign body ingestion. There were no adverse or unanticipated events reported during the follow-up period.

## 3 Discussion

This case report documents a rare presentation of small bowel obstruction (SBO) in a 7-month-old infant caused by the ingestion of a whole Shine-Muscat grape, a common but overlooked dietary risk. The primary strength of this case lies in its contribution to the limited literature on fruit ingestion as a cause of SBO in pediatric patients. Unlike the more frequently encountered foreign bodies, such as coins, magnets, or sharp objects, which are widely recognized and addressed in pediatric guidelines, fruits like grapes are often considered benign and are not typically associated with severe complications. This case emphasizes the need for pediatricians and caregivers to be aware that even seemingly harmless foods can pose a significant risk of gastrointestinal obstruction in young children. Given the rarity of this presentation, we reviewed the relevant literature to better understand the broader context of pediatric SBO.

A review of the relevant literature reveals that most cases of small bowel obstruction (SBO) in children are due to adhesions, intussusception, hernias, or congenital abnormalities (1). In contrast, SBO caused by foreign bodies is relatively rare, making up a small fraction of cases. These cases are most often associated with ingestion of hazardous or indigestible objects, such as magnetic balls, sharp objects, or superabsorbent materials (e.g., water-absorbing beads), all of which can lead to significant morbidity if not promptly treated (2–4, 8–14). In pediatric populations, SBO caused by food-related foreign bodies is even less common, with phytobezoars being one of the more frequent culprits (5, 7, 15–17). However, there is only one reported case of a newborn developing bowel obstruction due to grape pulp from ingested grape juice (18). Apart from this, there have been no documented cases of whole grapes causing bowel obstruction in young children. Pediatric cases involving whole fruits are particularly uncommon largely because these types of obstructions tend to occur in populations with predisposing factors, such as elderly patients with impaired swallowing or individuals with underlying gastrointestinal abnormalities (19, 20). A summary of various types of ingested foreign bodies leading to pediatric SBO, based on the available literature, is presented in Table 1 of the manuscript. These reports show that while phytobezoars and similar materials can occasionally cause obstruction, the ingestion of whole fruits presents unique anatomical and mechanical challenges, especially in young children with smaller lumens and immature swallowing mechanisms.

**Table 1:** Summary of pediatric small bowel obstruction cases caused by ingested foreign bodies from PUBMED in the past 20 years.

Type	Number of cases	Age	Presentation	Diagnosis	Complications	Imaging findings	Treatment
Magnetic objects (2–4)	3	7 yr, 10 yr, 18 mo	Abdominal pain, bilious vomiting, tenderness, abdominal distension	Small bowel obstruction due to multiple magnets	Intestinal necrosis, near perforation of ileum and caecum, bowel perforation, intra-abdominal abscess	x-ray: Radio-opaque objects in lower abdomen, dilated bowel loops, small bowel obstruction	Removal of magnets, resection and anastomosis, wedge excision, antibiotics (piperacillin/tazobactam)
Water-absorbing objects (8–12)	5	10 mo, 13 mo, 15 mo, 2 yr, 10 mo	Emesis becoming bilious, abdominal distension, constipation, air-fluid levels, non-bloody emesis later, hypoglycemia	Small bowel obstruction due to water-absorbing beads (Orbeez), superabsorbent toy ball	Hypoglycemia	x-ray: Distended small bowel, nonobstructive gas pattern; Ultrasound: Anechoic lesions in small bowel, anechoic cystic lesion; CT: Fluid-attenuation cystic mass	Surgical removal of Orbeez balls, whole bowel irrigation using polyethylene glycol-electrolyte, endoscopic crushing and removal of toy ball
Rubber or plastic objects (13, 14)	2	10 mo, 10 yr	Vomiting for 10 days, bilious vomiting, no bowel movement for 5 days, abdominal pain, distension	Small bowel obstruction due to rubber glove bezoar, rubber balls	None reported	x-ray: Dilated small bowel; CT: Distal small bowel obstruction due to bezoar	Surgical removal of rubber glove bezoar, rubber balls
Bezoars (plant/stone) (5, 7, 15–17)	6	3 mo, 4 mo, 11 yr, 16 mo, 5 yr, 1 yr	Vomiting, abdominal distension, irritable crying, acute abdominal pain, vomiting of brown fluid and undigested food, no stool or flatus for 3 days	Intestinal obstruction by potato bezoar, phytobezoar, trichobezoar	Recurrence after 8 months	x-ray: Dilated stomach, numerous air-fluid levels; Ultrasound: Echogenic image in bowel lumen; CT: Distended bowel, intraluminal mass	Endoscopic and surgical removal of bezoar, psychological therapy
Other foreign bodies (21–23)	3	22 mo, 12 yr, 7 wk	Bilious vomiting, colicky abdominal pain, distension, stool retention, copremesis	Small bowel obstruction due to pacifier nipple, coin at ileocecal valve, lactobezoar	None reported	x-ray: Dilated loops; Contrast enema: Distended proximal loops; Radiopaque shadow of coin, air-fluid levels; x-ray: Massively dilated intestinal loops	Laparoscopic removal of pacifier nipple, colonoscopic removal of coin, surgical removal of lactobezoar

SBO, small bowel obstruction; CT, computed tomography; DVT, deep vein thrombosis; yr, year(s); mo, month(s); wk, week(s); POI, postoperative intussusception; AER, air enema reduction.

Based on this literature review, we note that the case of a whole Shine-Muscat grape causing obstruction is a highly unusual instance. In our case, the smooth surface, large size, and indigestibility of the Shine-Muscat grape contributed to its lodgment in the small intestine, ultimately causing mechanical obstruction. This observation is consistent with isolated reports in adults where whole grapes have caused similar obstructions, primarily due to anatomical factors such as the size of the bowel lumen and the lack of sufficient peristaltic force to overcome the obstruction (19, 20). These findings emphasize the importance of including whole fruits as a potential risk factor for gastrointestinal obstruction, even in populations traditionally considered less susceptible, such as healthy children.

Moreover, our case demonstrates the clinical significance of integrating imaging findings, laboratory results, and patient history to make an accurate diagnosis. The lack of a clear ingestion history from the parents initially complicated the diagnostic process, highlighting the need for pediatricians to maintain a high index of suspicion for foreign body ingestion, even when common symptoms like vomiting and abdominal distension are the primary clinical signs. By relying on imaging, which demonstrated characteristic air-fluid levels and an intraluminal foreign body, the diagnosis of SBO became evident, guiding the subsequent minimally invasive intervention. Such cases underline the need for increased awareness among caregivers and healthcare providers about the risks posed by seemingly harmless foods like grapes, which can lead to severe complications if not properly managed.

The clinical decision-making in this case was guided by both the laboratory and imaging findings. The elevated C-reactive protein (CRP) and procalcitonin levels indicated an inflammatory response, possibly secondary to bowel obstruction. Though non-specific, the elevated CRP level further supports the presence of an inflammatory process. Meanwhile, the abdominal CT scan provided definitive evidence of a foreign body causing luminal obstruction. Despite the initial denial of foreign body ingestion by the parents, the diagnostic imaging suggested otherwise, underscoring the importance of correlating clinical, laboratory, and imaging data for accurate diagnosis. The successful use of a minimally invasive laparoscopic approach to crush and mobilize the grape into the colon, rather than performing an enterotomy, minimized surgical trauma and promoted a faster recovery, consistent with current surgical best practices.

The limitations of this case report include its singular nature, which limits the generalizability of the findings to broader pediatric populations. However, it serves as a valuable reminder that common dietary items can pose unforeseen risks. Moreover, the report's strength is its detailed documentation of a novel management approach for a rare cause of pediatric SBO, potentially guiding future cases with similar presentations.

In conclusion, based on the above discussion, this case reinforces several important takeaways for clinical practice. First, it is crucial to consider less common etiologies, such as fruit ingestion, when evaluating pediatric patients with gastrointestinal symptoms, particularly when typical causes are excluded. Second, this report underscores the importance of preventive education for caregivers on safe feeding practices, such as cutting grapes into smaller pieces or avoiding whole grapes altogether for young children. Finally, adopting minimally invasive surgical techniques where feasible can reduce patient morbidity and lead to better outcomes, as evidenced by the rapid recovery in this case.

## Data Availability

The datasets presented in this article are not readily available because The dataset is restricted to use for research purposes only and cannot be shared with unauthorized individuals. Access is limited to personnel directly involved in the study, and all data must be anonymized to protect patient confidentiality. Requests to access the datasets should be directed to zhangyoucheng2005@126.com.
